# The prevention and management of chronic disease in primary care: recommendations from a knowledge translation meeting

**DOI:** 10.1186/s13104-015-1514-0

**Published:** 2015-10-15

**Authors:** Sara Ahmed, Patrick Ware, Regina Visca, Celine Bareil, Maud-Christine Chouinard, Johanne Desforges, Roderick Finlayson, Martin Fortin, Josée Gauthier, Dominique Grimard, Maryse Guay, Catherine Hudon, Lyne Lalonde, Lise Lévesque, Cecile Michaud, Sylvie Provost, Tim Sutton, Pierre Tousignant, Stella Travers, Mark Ware, Amede Gogovor

**Affiliations:** Faculty of Medicine, School of Physical and Occupational Therapy, McGill University, 3654 Prom Sir-William-Osler, Montreal, QC H3G 1Y5 Canada; Constance-Lethbridge Rehabilitation Center, Centre de recherche interdisciplinaire en réadaptation (CRIR), 7005 Boulevard De Maisonneuve O, Montréal, Quebec H4B 1T3 Canada; Clinical Epidemiology, McGill University Health Center, 687 Pine Ave W, Montreal, QC H3A 1A1 Canada; Centre for Expertise in Chronic Pain of the Réseau universitaire intégré de santé McGill, 2155 Guy, Montreal, QC H3H 2R9 Canada; Department of Management, HEC Montreal, 3000, chemin de la Côte-Sainte-Catherine, Montreal, QC H3T 2A7 Canada; Département des sciences de la santé, Université du Québec à Chicoutimi, 555 boul. de l’Université, Chicoutimi, QC G7H 2B1 Canada; Centre de santé et de services sociaux de Chicoutimi, 305 St-Vallier, Chicoutimi, QC G7H 5H6 Canada; Groupe de médecine de famille de Verdun, 4000, boulevard Lasalle, Verdun, QC H4G 2A3 Canada; Département de médecine de famille et médecine d’urgence, Faculté de médecine et des sciences de la santé, Université de Sherbrooke, 3001 12ème avenue Nord, Fleurimont, QC J1H 5N4 Canada; Institut national de santé publique du Quebec, Consortium InterEst Santé, Département des sciences infirmières, Université du Québec à Rimouski, 300 Allée des Ursulines, Bureau K-310, Rimouski, QC G5L 3A1 Canada; Direction de santé publique de l’Agence de la santé et des services sociaux de Montréal, 1301 Sherbrooke Est, Montreal, QC H2L 1M3 Canada; Département des sciences de la santé communautaire, Faculté de médecine et des sciences de la santé, Université de Sherbrooke, 3001 12e Avenue Nord, Sherbrooke, QC J1H 5N4 Canada; Direction de santé publique de l’Agence de la santé et des services sociaux de la Montérégie, 1255 Beauregard, Longueuil, J4H 2M3 Canada; Faculté de pharmacie, Université de Montréal, pavillion Jean-Coutu, Montreal, QC H3C 3J7 Canada; Centre de santé et de services sociaux de Laval, 1755 boulevard René-Laennec, Laval, QC H7M 3L9 Canada; Faculté de médecine et des sciences de la santé, École des sciences infirmières, Université de Sherbrooke, Campus de Longueil, 150, Place Charles LeMoyne, Bureau 200, Sherbrooke, QC J4K 0A8 Canada; Centre de santé et de services sociaux du Roché-Percé, 451, rue MGR-Ross Est, C.P. 3300, Chandler, QC G0C 1K0 Canada; Alan Edwards Pain Management Unit of the McGill University Health Centre, 650 Cedar Avenue, Montreal, QC H3G 1A4 Canada

**Keywords:** Chronic disease management, Knowledge translation, Implementation, Evaluation

## Abstract

**Background:**

Seven chronic disease prevention and management programs were implemented across Quebec with funding support from a provincial-private industry funding initiative. Given the complexity of implementing integrated primary care chronic disease management programs, a knowledge transfer meeting was held to share experiences across programs and synthesize common challenges and success factors for implementation.

**Methods:**

The knowledge translation meeting was held in February 2014 in Montreal, Canada. Seventy-five participants consisting of 15 clinicians, 14 researchers, 31 knowledge users, and 15 representatives from the funding agencies were broken up into groups of 10 or 11 and conducted a strengths, weaknesses, opportunities, and threats analysis on either the implementation or the evaluation of these chronic disease management programs. Results were reported back to the larger group during a plenary and recorded. Audiotapes were transcribed and summarized using pragmatic thematic analysis.

**Results and discussion:**

Strengths to leverage for the implementation of the seven programs include: (1) synergy between clinical and research teams; (2) stakeholders working together; (3) motivation of clinicians; and (4) the fact that the programs are evidence-based. Weaknesses to address include: (1) insufficient resources; (2) organizational change within the clinical sites; (3) lack of referrals from primary care physicians; and (4) lack of access to programs. Strengths to leverage for the evaluation of these programs include: (1) engagement of stakeholders and (2) sharing of knowledge between clinical sites. Weaknesses to address include: (1) lack of referrals; (2) difficulties with data collection; and (3) difficulties in identifying indicators and control groups. Opportunities for both themes include: (1) fostering new and existing partnerships and stakeholder relations; (2) seizing funding opportunities; (3) knowledge transfer; (4) supporting the transformation of professional roles; (5) expand the use of health information technology; and (6) conduct cost evaluations. Fifteen recommendations related to mobilisation of primary care physicians, support for the transformation of professional roles, and strategies aimed at facilitating the implementation and evaluation of chronic disease management programs were formulated based on the discussions at this knowledge translation event.

**Conclusion:**

The results from this knowledge translation day will help inform the sustainability of these seven chronic disease management programs in Quebec and the implementation and evaluation of similar programs elsewhere.

**Electronic supplementary material:**

The online version of this article (doi:10.1186/s13104-015-1514-0) contains supplementary material, which is available to authorized users.

## Background

There is consensus that meeting the challenges of the increasing prevalence of chronic disease with its impacts on the health system and patient quality of life will require major changes in the health system. In Canada, healthcare reform is driven by the increasing burden of wait times, comorbidities and cost among others, and has involved stakeholders at local, provincial, and the federal level. This resulted in a number of initiatives and commitments across the country, including the commitment of provincial premiers in 2004 on the future of health care [1–6].

At the provincial level, the *Ministère de la Santé et des Services sociaux* (MSSS) of Quebec has made the prevention and management of chronic diseases a priority by developing and disseminating a strategy for the prevention and management of chronic diseases to all health and social services agencies (ASSS) and by mobilizing the Fonds de recherche Québec—Santé (FRQS), the provincial health research funding agency for peer-review competitions. The intended purpose was to recognize and support best practices and their integration into a continuum of services, and to mobilize all stakeholders concerned with chronic diseases around the following objectives and outcomes: (1) reduce the risk factors that contribute to chronic disease; (2) reduce the complications of chronic diseases; (3) reduce hospitalizations and emergency stays for people with chronic diseases; (4) improve the use of drugs; (5) improve patients’ quality of life and satisfaction with chronic disease prevention and management programs as well as the satisfaction of those who care for them; (6) provide individuals with self-management support; (7) improve the satisfaction of professionals in their daily clinical practice; and (8) improve the health of the population [7].

### The Pfizer-FRQS-MSSS initiative for the prevention and management of chronic disease

The *Fonds Pfizer*-*FRQS*-*MSSS sur les maladies chroniques* fund was established in 2011 to support innovative projects, driven by local health and social services in collaboration with health researchers. This two-part program was set up to fund projects aimed at implementing initiatives related to the prevention and management of chronic diseases (clinical component), and evaluation of these initiatives (evaluative component).

Funded projects needed to meet the following conditions: (1) submitted by one or more health and social services centers (CSSS) in collaboration with one or more family physicians practicing at the primary care level, and one or more researchers in the field of health and social services; (2) promote a patient-centered interdisciplinary approach for the management of individuals with chronic diseases at an intensity appropriate to their health conditions, and/or promote the development of tools and approaches for self-management support; (3) include a process for formal scientific review so that factual data and evidence can be gleaned at various stages of the project, and so that the efficacy, productivity and/or efficiency of the proposed approaches or courses of actions can be assessed. The evaluation should also produce knowledge and lessons for other clinical settings that may wish to implement the initiatives or tools developed [8].

To date seven projects were funded. The current report aims to summarize a 1 day knowledge transfer meeting organized to share preliminary results and discuss ways to transfer the knowledge gained to other CSSS, and ASSS, and maximize the use of evidence from the Quebec context. The agenda of the meeting (see Additional file 1) included the presentation of the programs followed by small group discussions around two themes: (1) success factors and challenges of the implementation of chronic disease management and prevention programs, and (2) lessons learned from the evaluation of the implementation and effectiveness of each program. The following report presents an overview of each of the projects, highlighting similarities and differences between them to provide context to the results of the round table discussions. The overarching objective was to highlight the common experiences and generate key messages to optimize the implementation and evaluation of chronic disease management programs.

### Characteristics of the seven programs

The evaluation and selection of the research proposals were carried out by the *Fonds de recherche du Québec*—*Santé* (FRQS), the provincial funding agency. The seven funded programs included are listed below. Details of each program are found in Table 1.

**Table 1:** Characteristics of the of the Pfizer-FRQS-MSSS funded programs

Program	Sites	Target population	Health professionals in interdisciplinary team	Basis for self-management program	Study design	Evaluation method
P1. Implementation and evaluation of an integrated primary care network for prevention and management of chronic pain	4 CSSS	Inclusion: low back pain <1 year, exclusion: severe dependence, mental of cognitive problems; Covered by CSST or SAAQ	Doctor, nurse, psychologist, physiotherapist	Stanford model 5A program	Quasi-experimental multiple pretest/posttest interrupted time series	Mixed methods (triangulation): qualitative (multiple case study) and quantitative
P2. Evaluation of an integrated primary care network for prevention and management for cardiometabolic risk in Montreal	6 CSSS	Inclusion: marginal fasting glycemia, or glucose intolerance, or diabetes treated with diet only, or diabetes treated with monotherapie, or diabetes treated with more than one medication if HbA1c ≤8.0 %; as well as adults with hypertension with BP in the doctor’s office ≥140/90 (if diabetic, with BP ≥130/80)	Nurse, psychologist (or social worker), kinesiologist, nutritionist, pharmacist		Quasi-experimental multiple pretest/posttest interrupted time series	Mixed methods (triangulation): qualitative (multiple case study) and quantitative
P3. TRANSforming InTerprofessional clinical practices to improve cardiovascular disease prevention in primary care [10, 11]	8 FMG	Inclusion: multimorbid patients with moderate to high cardiovascular risk	Doctor, nurse, pharmacist, and either a nutritionist, kinesiologist, or psychologist		Randomized trial NCT01418716	Development: Participatory research
						Impact: qualitative and quantitative, with triangulation
P4. SIID2: intersectoral and interdisciplinary management of type 2 diabetes (http://www.siid2.ca)	1 RLS	Inclusion: 45 years+, all patients at-risk of diabetes (screened with CANRISK) or with diabetes	Doctor, nurse, kinesiologist, social worker, nutritionist, pharmacist,	CCM	Randomized trial	Quantitative (impact and implementation)
					Survey	
		Exclusion: Pregnancy, institutionalized patients, cognitive deficit				
P5. PR1MaC: evaluating the integration of chronic disease prevention and management services into primary health care [12]	2 CSSS	Inclusion: patients aged 18 and 75 years with at least one of the following conditions: diabetes, cardiovascular disease, COPD, asthma or risk factors (smoking, obesity, dyslipidemia, glucose intolerance, and metabolic syndrome, sedentarity)	Nurse, kinesiologist, nutritionist, smoking cessation therapist, respiratory therapist	CCM, Stanford	Randomized trial with delayed intervention arm, before-and-after design with repeated measures, and quasi-experimental design using a comparative cohort NCT01319656	Realist evaluation and practical participatory approach (implementation)
						Quantitative (impact)
		Exclusion: patients with serious cognitive problems				
P6. VISAGES: implementation and evaluation of a pragmatic intervention of case management and self-management support for frequent users [13]	2 CSSS	Inclusion: high users of hospital services, aged 18–80 years with diabetes, cardiovascular disease, respiratory diseases, musculoskeletal diseases and/or chronic pain	Doctor, nurse, psychologist, social worker, kinesiologist, nutritionist, pharmacist	Stanford model	Implementation analysis, Randomized trial with delayed intervention arm NCT01719991	Realist evaluation and practical participatory approach (implementation)
		Exclusion: severe mental health or cognitive problems				
	4 FMG					
						Qualitative and quantitative (impact)
						Cost-effectiveness and cost-benefit analysis
					Economic analysis	
P7. Self management of health within the territory of Rocher-percé	2 CSSS	Inclusion: patients aged 18 and over with ≥1 chronic conditions (diabetes, COPD, cardiovascular disease, kidney failure) and related risk factors (obesity, hypercholesterolemia, HT etc.)	Nurse, kinesiologist, nutritionist	Home made	Quasi-experimental multiple pretest/posttest interrupted time series	Collaborative research with developmental approach
						Mixed methods for efficacy (triangulation): qualitative and quantitative
						Qualitative (implementation)
		Exclusion: severe dependence, cognitive problems, decompensated cardiac insufficiency, stage 3–4 COPD, Severe uncontrolled HT				

*CSSS* health and social service centres, *FMG* family medicine group, *CSST* health and occupational safety commission, *SAAQ* automobile insurance society of Quebec, *COPD* chronic obstructive pulmonary disease, *RLS* local service network

- *P1* Implementation and evaluation of an integrated primary care network for prevention and management of chronic pain
- *P2* Evaluation of the implementation of an integrated primary care network for prevention and management of cardiometabolic risk in Montréal [9]
- *P3* TRANSforming InTerprofessional clinical practices to improve cardiovascular disease prevention in primary care [10, 11]
- *P4* Intersectoral and interdisciplinary management of type 2 diabetes
- *P5* Adaptation, implementation and evaluation of rehabilitation services for chronic disease prevention and management integrated into primary health care [12]
- *P6* Implementation and evaluation of a pragmatic intervention of case management and self-management support for frequent users of hospital services with chronic diseases [13]
- *P7* Self-management of health within the territory of Rocher-Percé.

### Conceptual framework

The Chronic Care Model (CCM), an evidence-based framework for the management of chronic diseases that describes the interaction between the health care setting, community and patient as they relate to health outcomes, is informing the implementation of disease management and prevention strategies across Canada. It has been adopted by the MSSS’s strategy for the prevention and management of chronic diseases. All but one program was directly based on the CCM. P6 was based on the model of the UK National Health Service on innovation in health care and social services for people with CD [14]. “This model incorporates the basic principles of the Chronic Care Model [15, 16], while also drawing on lessons learned from US models, such as that of Kaiser Permanente, with regard to the intensity of care that is appropriate for the complex needs of patients [17], and of the Evercare model [18], with regard to the use of case management nurses in primary care” [13]. Table 2 presents the empirically supported components of the CCM, with an indication of the components applied by each of the programs.

**Table 2:** Elements of Chronic Care Model (CCM) included in each program

CCM component	P1	P2	P3	P4	P5	P6	P7
*Health system*							
Visibly support improvement at all levels of the organization, beginning with the senior leader					√		√
Promote effective improvement strategies aimed at comprehensive system change						√	√
Encourage open and systematic handling of errors and quality problems to improve care	√	√				√	√
Provide incentives based on quality of care							
Develop agreements that facilitate care coordination within and across organizations	√	√		√	√	√	√
*Delivery system design*							
Define roles and distribute tasks among team members	√	√	√	√	√	√	√
Use planned interactions to support evidence-based care	√	√	√	√	√	√	√
Provide clinical case management services for complex patients			√			√	√
Ensure regular follow-up by the care team	√	√	√	√	√	√	√
Give care that patients understand and that fits with their cultural background				√	√	√	√
*Decision support*							
Embed evidence-based guidelines into daily clinical practice	√	√	√	√	√	√	√
Share evidence-based guidelines and information with patients to encourage their participation	√	√	√	√	√	√	√
Use proven provider education methods	√	√	√	√	√		√
Integrate specialist expertise and primary care	√			√	√	√	√
*Clinical information systems*							
Provide timely reminders for providers and patients	√	√				√	√
Identify relevant subpopulations for proactive care	√	√	√			√	√
Facilitate individual patient care planning	√	√	√	√	√	√	√
Share information with patients and providers to coordinate care	√	√	√	√	√	√	√
Monitor performance of practice team and care system	√	√		√		√	√
*Self-management support*							
Emphasize the patient’s central role in managing their health	√	√	√	√	√	√	√
Use effective self-management support strategies that include assessment, goal-setting, action planning, problem-solving and follow-up	√	√	√	√	√	√	√
Organize internal and community resources to provide ongoing self-management support to patients	√	√	√	√		√	√
*The community*							
Encourage patients to participate in effective community programs	√	√	√	√	√	√	√
Form partnerships with community organizations to support and develop interventions that fill gaps in needed services		√		√	√	√	√
Advocate for policies to improve patient care	√				√		√

### Program clinical process and interventions

All of the programs use different combinations of interventions to achieve their specific goals. The interventions in programs P1, P2 and P4 have many similarities; they are based on interdisciplinary team care, standardized clinical process, support for primary care physicians and for patient self-management support, and coordination of care across levels. P4 also includes ‘social marketing’ for primary care physicians and online training for pharmacists.

In contrast, a clinician, acting as point person, plays a central role in the programs P3 and P5. In P3, the primary care nurse performs the evaluation of the cardiovascular health and ensures the referral of the patients to other health professionals. P3 also included an intervention, facilitation, specifically developed to support program implementation and included the provision of internal facilitators represented by clinicians from family medicine groups (FMG) and external facilitators. Interventions for P5 include nurse led interventions for self-management support, education on diseases (diabetes, COPD, asthma, cardiovascular), education on risk factors (pre-diabetes, high blood pressure, dyslipidemia, obesity, physical inactivity, smoking), motivational interviewing, education about nutrition, education about physical activity, and counselling on medication use and on smoking cessation.

P7 is organised around a kinesiologist, nurse and nutritionist (K-I-N triad), who as a team provide interdisciplinary management and follow-up for individuals with chronic diseases in the Rocher-Percé territory. The primary care nurse holds the pivotal roles of medical and pharmacological monitoring and coordination with family physicians and specialised services. P6 is a very case-management oriented program with case management by FMG nurses who are charged with: (1) evaluation of the patient’s needs and resources; (2) establishment and maintenance of a patient-centred, individualized service plan; (3) coordination of services among partners; and (4) self-management support for patients and families. All patients are given the opportunity to participate in self-management groups based on the Stanford program [19].

### Evaluation study design

#### Program implementation

To evaluate the implementation of the programs, three programs used mixed methods (P1, P2, P3), one program used a survey method (P4), and one program (P7) used only qualitative methods. Finally, two programs undertook a realist evaluation and practical participatory approach to evaluate the program’s implementation (P5, P6).

#### Impact on patient outcomes

The process for evaluating the impact on patient outcomes was based on a combination of qualitative and quantitative methods in the majority of the programs. Three programs (P1, P2 and P7) specifically adopted the mixed methods approach with triangulation of the data and a quasi-experimental before-after design with repeated-measures for patient impact evaluation. A randomized design was used for four programs (P3, P4, P5 and P6) and a quasi-experimental before-after design with repeated-measures was used for three programs (P1, P2 and P5) with a comparison group for one program (P5). Various qualitative methods were reported by the programs and included participatory and multiple case study approaches. P6 also conducted an economic analysis looking at cost-effectiveness and the cost-benefit of implementing the program.

### Outcome measures

#### Implementation

Table 3 presents the variety of elements considered when evaluating the implementation of the programs; these elements were grouped into broader categories. While a common implementation evaluation model did not exist among the seven programs, in general, each considered most or all of the elements of each domain. Two exceptions exist. The first is that P5 and P6 did not assess support offered to healthcare professionals in the program as part of the implementation evaluation, and the second is that only two programs (P3 and P6) initially set out to assess the cost of implementation.

**Table 3:** Implementation measures

Domain evaluated	Elements considered	P1	P2	P3	P4	P5	P6	P7
Resources	Community and potential partner organizations in the region	✓	✓		✓	✓	✓	✓
	Human	✓	✓	✓	✓	✓	✓	✓
	Financial	✓	✓	✓	✓	✓	✓	
	Physical space	✓	✓		✓	✓	✓	✓
	Strategies and approaches (ex. CME)	✓	✓	✓	✓	✓	✓	
	Information sharing	✓	✓	✓	✓	✓	✓	✓
Organizational structure	Decision making roles	✓				✓		
	Remuneration of health professionals	✓				✓		
	Consultation structure within the program	✓	✓	✓	✓	✓		
	Establishing links with partners	✓	✓	✓	✓	✓	✓	
	Role of stakeholders in the success of the program	✓	✓	✓	✓	✓	✓	✓
	Follow-up by program clinicians	✓		✓	✓	✓	✓	✓
Reach within target population	Patients	✓	✓	✓	✓	✓	✓	✓
	Referring health professional	✓	✓	✓	✓	✓	✓	✓
	Medical clinics	✓	✓	✓	✓	✓	✓	✓
	CSSS		✓		✓			
Support for referring HP	Continuing medical education sessions	✓	✓	✓	✓		✓	
	Development of clinical tools and forms	✓	✓	✓	✓	✓	✓	✓
	Communications with referring health professionals	✓	✓	✓	✓	✓	✓	✓
Support for program HPs	Formal and informal training sessions	✓	✓	✓	✓			
	Regional professional committees		✓		✓			
Contextual facilitators and barriers	Clinician and stakeholder incentives	✓	✓	✓		✓		
	Change management strategies	✓	✓	✓		✓	✓	✓
	Confidence and engagement of stakeholder	✓	✓	✓		✓	✓	✓
	Organizational structure of the clinic	✓	✓	✓		✓	✓	
	Organizational structure of services within CSSS	✓	✓		✓	✓	✓	✓
	Building collective knowledge and leadership	✓	✓	✓	✓	✓	✓	✓
	Appropriation by stakeholder		✓	✓				
	CSSS external environment		✓		✓			
Waiting time	Delay between reception of the referral to program and the 1^st^ visit	✓				✓	✓	
Impact on Primary care	Participation in the program	✓	✓	✓	✓	✓	✓	✓
	Physicians perception of impact on patients	✓	✓	✓	✓	✓	✓	✓
	Interprofessional collaboration	✓	✓	✓	✓			✓
	Perception benefit of the program	✓	✓	✓	✓	✓	✓	✓
	Improvement of knowledge regarding management of patient with chronic condition and resources available	✓	✓	✓	✓	✓	✓	
	Use of CCM components and clinical tools	✓	✓	✓	✓	✓		✓
	Doctors participating in CME sessions	✓			✓			
	Management of individuals with chronic disease (ACIC)	✓			✓			✓
Cost	Cost-effectiveness			✓			✓	
	Cost-benefit						✓	

*HP* health professionals, *CME* continuing medical education, *CSSS* centre de santé et de services sociaux, *ACIC* assessment of chronic illness care

#### Impact on patient outcomes

Table 4 presents measures for the impact evaluations for each program. While the specific measurement tools used were specific to the chronic disease targeted by the program, many of the programs measured similar outcomes. The impact measures in Table 4 are categorized into three sections: patient reported outcomes, clinical outcomes, and use of healthcare services. In terms of patient reported outcomes, a wide range of measures are used by one or more of the programs to evaluate the following constructs: pain intensity and interference, physical function, anxiety, depression, quality of life, self-management, management of chronic disease, psychological distress, social isolation, literacy, patient activation, lifestyle habits, and satisfaction with care. Many of the blood and physical clinical measures are common among four of the seven programs (P2, P3, P4, P7) and all seven programs assess use of healthcare services.

**Table 4:** Patient impact measures

Construct	Measure	P1	P2	P3	P4	P5	P6	P7
*Patient reported outcomes*								
Pain intensity and interference	Brief pain inventory	✓						
Physical function	Oswestry	✓						
Anxiety	HADS	✓					✓	
Depression	HADS	✓					✓	
	PHQ-9	✓						
Self-efficacy	Self-efficacy scale	✓						✓
	SEM-CD					✓	✓	
Quality of life	SF-36							✓
	SF-12	✓				✓	✓	
	ADDQoL		✓					
Level of risk	Keele start back	✓						
Self-management (empowerment)	HeiQ	✓				✓	✓	
	SDSCA		✓					
	PIH				✓			
Management of chronic disease	PACIC	✓	✓	✓	✓			✓
Co-morbidity	DBMA					✓	✓	
Psychological distress	(K-6)					✓	✓	
Social isolation	Nottingham health profile						✓	
Literacy	NVS						✓	
Patient activation	PAM						✓	✓
Lifestyle habits	Physical activity		✓	✓		✓	✓	✓
	Smoking		✓	✓		✓	✓	✓
	Eating habits		✓	✓		✓	✓	✓
Satisfaction with care	Interview/questionnaire	✓	✓			✓	✓	✓
	Care experience survey	✓			✓			✓
*Clinical measures*								
Blood	HbA1C		✓	✓	✓			✓
	Blood pressure		✓	✓	✓			✓
	LDL-C		✓	✓	✓			
	Glycemia		✓	✓	✓			✓
	Lipid profile		✓	✓				✓
Physical	Waist size		✓	✓	✓			✓
	BMI		✓	✓	✓	✓		✓
Use of healthcare services								
Patient use of services	ER visit	✓	✓		✓	✓	✓	✓
	Hospitalization	✓	✓	✓	✓	✓	✓	✓
	Visits to other professionals		✓	✓	✓	✓	✓	✓

*HADS* Hospital Anxiety and Depression Scale, *PACIC* patient assessment of chronic illness care, *PIH* partner in health, *ADDQoL* audit of diabetes dependent quality of life tool, *SEM-CD* self-efficacy for managing chronic disease, *SDSCA* summary of diabetes self-care activities measure, *DBMA* the disease burden morbidity assessment, *K-6* Psychological Distress Scale, *PAM* patient activation measure, *NVS* newest vital sign

## Methods

### Round table discussions

Each project team selected at least two members to attend the meeting. Participants (n = 75) were a blend of 15 clinicians, 14 researchers, 31 knowledge users [government decision makers (n = 25), clinical administrators (n = 3), university and academic health network members (n = 3)], and representatives from the funding agencies [Pfizer (n = 5), MSSS (n = 6), and FRQS (n = 4)] (see Additional file 2). Verbal consent was obtained by all participants at the commencement of the meeting proceedings. Each of the participants was assigned to one of seven round tables by the organizers of the event in order to ensure equal representation of stakeholders and members of each project team. Each round table, consisting of 10–11 participants, was assigned one of two themes to consider. Each group was also assigned a facilitator who guided the discussions and a rapporteur who took notes. At the end of the group discussion it was the rapporteur who presented a summary of the round table discussions during the larger group session. Each round table was asked to identify the strengths, weaknesses, opportunities, and threats (SWOT) regarding the themes. The larger group report-back session and discussion was audio-taped and transcribed. In the week following the meeting, two independent reviewers (AG and PW) conducted a pragmatic thematic analysis of the transcripts [20]. Themes were reviewed and refined by the working committee leading the writing of the report (SA, RV, AG, PW). The results of the SWOT analysis and conclusions that stemmed from this analysis were validated with members of the follow-up committee, which consisted of members from each of the funded projects. The committee reviewed the initial version of the synthesis of the SWOT analysis, and comments were integrated. The document was circulated until minimal suggested revisions were proposed. This knowledge translation meeting, and the publication of its results, was exempted from requiring ethics committee approval by the McGill University Health Centre Research Ethics Board.

## Results and discussion

Results of the SWOT analysis for the theme of success factors and challenges of implementing chronic disease prevention and management programs are found in Table 5. Results for the SWOT analysis of Theme 2, opportunities for cross comparison of results across the projects and remaining questions to determine effectiveness of the projects, are found in Table 6. Even though each round table was given different themes to consider, given the interdependency between implementation and evaluation of the programs, some of the SWOT areas were repeated in Theme 1 and 2. In Tables 5 and 6 we placed the majority of factors according to whether they were most closely related to implementation or evaluation. However, we elected to retain some overlap between the two tables when we felt that the repeated areas held different implications depending on whether it is viewed within the context of implementation or evaluation.

**Table 5:** Results of SWOT analysis for the implementation of chronic disease prevention and management programs

Strengths to leverage	Weaknesses to address
*Stakeholders, partnerships, and knowledge transfer*	*Funding*
Having a “complete picture” and understanding [needs] of key stakeholders, including decision- makers and their willingness to support change	Length of project is too short to make a clinical or behavioural change in patients
Strong government leadership (local, regional, and national)	Evaluation component is under financial and evaluation constraints bringing delays in patient interventions
*Clinicians and clinical teams*	Lack of resource and funds (local, regional, and national)
Motivated clinicians Immediate embracing of the program by some clinicians—willingness to go above and beyond what the pilot project was meant to implement	Funding required sites to commit to the longevity of programs before programs were proven to be effective
	*Communication* Lack of communication/marketing plan aimed at reaching target populations
The presence of complete clinical teams composed of various professionals allows clinicians to learn from one another	
Strong clinical leadership in the diverse professions	Ineffective communication of teams in primary care and no systematic communication with referring doctors
*Program structure*	*Clinicians and clinical teams*
A common model of care between projects Ability of a program to integrate into existing structures. Projects need to be able to weave a place into what already exists	Have a tendency to use “champion clinicians”. There is a danger in counting on “champions” who are not always available
	Existing medical culture closed to the concept of interdisciplinary and preventative interventions
Nature of the programs is evidence-based	Recruitment and turnover of personnel is especially difficult in a perspective of trying to transform clinical roles
Address diseases as well as their risk factors	Lack of participation from referring physicians
Patient-centered approach compared to a typical silo approach	*Program structure*
Touch on psychosocial factors as much as biological factors	Many tools available make the decision about choosing which one to use difficult
Emphasis on interdisciplinary teams, and self-management	Not having clinical information systems
	Low number of referrals to the programs. May be due to lack of awareness of referral forms or clear referral procedures
	Method of physician remuneration
	Lack of continuity of care

Opportunities to optimize	Threats to mitigate
*Funding*	
Seize public and private funding opportunities	*Funding*
Capitalize on existing funding to add resources to Family Medicine Groups	Ensure continuation of funding
Explore what currently exists in terms of remuneration models	Lack of resources and difficulty of resources management
*Stakeholders, partnerships, and knowledge transfer*	Ensuring constant and continuous data collection
Facilitate intra and inter-professional meetings for knowledge exchange and ensuring human contact between stakeholders	*Stakeholders, partnerships, and knowledge transfer* Integration of many organizations with different business models and cultures—brings challenges in terms of communication, authority, financing, etc
Working with people and community networks who have a shared vision and philosophy Influence changes in university curriculum to put greater emphasis on interdisciplinarity	
	Roles and responsibilities described in the law do not necessarily translate into real power or influence
Continuously talk about chronic disease in its entirety to incite others to associate themselves with the cause	Lobby presence (ex. pharmaceutical, professional federations) Mobilization of primary care teams and all the financial factors involved—resource re-allocation
*Clinicians and clinical teams* Make better use of clinical tools, stakeholders experience, and models which have already proven themselves—avoid reinventing the wheel	
	Will take effort to compile and disseminate the results of seven projects so that they can be used to guide decisions across the province
Capitalize on the synergy between research team and clinicians	*Clinicians and clinical teams*
Strong evaluations and the guidance it can provide throughout the implementation process and for guiding future decisions	Potential resistance that arises during the evaluation of clinical practice Harmonizing the visions in the management of chronic disease (ex. expert clinician vs. the patient partner)
Support the transformation of professional roles by exploring different types of training	
*Program structure*	Learning to work in interdisciplinary teams
Support integration of self-management into patient care	Lots of sudden changes can become tiresome
Adoption of health information technologies to facilitate referrals, care delivery, access to medical information, and communication	Ensure that professionals are using their full potential, particularly in a context of the revision of roles
Would be good to have access to a single tool that could facilitate work of the doctor and properly identify the patient’s needs and show where they are in their care path	Avoiding a doubling of services—make sure projects are not competing with existing services on a territory Physicians often lack a complete health profile of their clientele
Restructure programs to better respond to personnel turnover	
	A large proportion of the population does not have a family physician and therefore no access to these projects
	Conservative leadership of authorities—is the leadership sufficient to bring about the desired changes?

**Table 6:** Results of SWOT analysis for evaluating and communicating the effectiveness of chronic disease management programs

Strengths to leverage	Weaknesses to address
*Stakeholders, Partnerships, and Knowledge Transfer of evaluation results* Legitimate interest of potential community partners e.g. pharmacists Sincere interest in the program on the part of referring doctors, stakeholders, and clinicians The evaluation provides a natural feedback mechanism. Patients who see an improvement in their health bring this information back to their family doctors *Evaluation process* Similarity of programs (all based on CCM) Similarity of tools allows for the pooling of results to increase sample sizes and allow for comparison	*Funding* Lack of resources for evaluation *Communication* Lack of referrals to the program and difficulty in reaching the target population (small sample size)—expressed as a lack of marketing and communication skills Communication procedure are not well defined: need a mechanism to feed back patient outcomes to physicians and for them to communicate with the program to ask questions and see how their patient is doing *Stakeholders, partnerships, and knowledge transfer of evaluation results* Physicians not convinced about the efficacy of an interdisciplinary approach Difficulty in legitimizing the projects to key actors, often family doctors Difficulty in feeding back information about evaluation results to clinicians *Evaluation process* Different indicators between projects Need to establish key minimal indicators that must be collected throughout implementation Evaluation timeline too short Short timeline means incomplete implementation Lost participation leading to missing data Difficulty involving clinicians in the data collection process The act of evaluating is seen as an intervention in itself. Good for implementation but might introduce bias for evaluation Unclear definition of chronic diseases (ex. is cancer a chronic disease?) Cannot ignore notion of cost-benefits as many stakeholders are interested in knowing about the long-term feasibility at the institution level Lack of technology-supported tool All stakeholders have different things they want to measure, achieve, and evaluate. This is hard to consolidate

Opportunities to optimise	Threat to mitigate
*Stakeholders, Partnerships, and Knowledge Transfer of evaluation results*	*Funding*
Improve involvement of family doctors to ensure their participation and ensure their sense that they have a role to play	Not enough funding for an evaluation to prove a program’s long-term effectiveness, which is needed to seek more funding
Integration with local and community resources (ex. YMCA, pharmacists, gyms, kinesiologists)—need to get creative in terms of partnerships Make use of evaluation results to legitimize the projects to seek out funding and attract involvement of more people	Planning and financing of research—not enough resources to see the evaluation all the way through (implementation, evaluation, diffusion of results)
	*Stakeholders, partnerships, and knowledge transfer of evaluation results* Not enough publication of results to help decision makers *Evaluation process* Danger of saturating certain areas with too many similar projects
Synthesize the facilitating factors and challenges experienced by all projects	
Pursue and create new partnerships by bringing down barriers with the community	
Utilize existing resources like local regional tables to promote important networking opportunities	
Bring professionals together to standardize care and information given to patients	While there are similarities in evaluation tools, this isn’t always the case—it will be a challenge to harmonize tools and projects in the future
*Communication* Improve marketing and communication (ex. work with students). This can help bring awareness to the project and help researchers disseminate results	Clinical information systems could help in data collection—these are not available for most projects
	Obtaining an adequate control group Resistance to the evaluation of programs and of quality of care
Improve working conditions for professionals to incite them to participate in the program	
*Evaluation process*	
Need to evaluate costs - both cost of implementation and cost- effectiveness of project	
Do better job of evaluating physician dropout rates	

### Theme 1: success factors and challenges of the implementation of chronic disease prevention and management programs

Specific question for Theme 1: What are the key success factors and challenges of implementing chronic disease management and self-management programs, within the context of the seven chronic disease management programs? How can we ensure the sustainability of chronic disease management programs? Identify the strengths, weaknesses, opportunities and threats.

### Strengths to leverage

#### Synergy between clinical and research teams

One of the most important strengths discussed was the funding structure (clinical implementation and evaluation component) that allowed for a synergy between the research and clinical teams to evolve. These two groups working together ensure that a data collection process, based on clinically relevant indicators, can take place and be efficiently fed back to clinical teams. This synergy is reinforced by communication both within and between the respective interdisciplinary teams.

#### Stakeholders working together

Another strength identified was the motivation and mobilizations of all stakeholders including healthcare professionals, patients, managers, members from the CSSS, the ASSS and MSSS, and potential collaborators in the private sector. Mobilization itself is made easier if there is an intimate understanding of the needs of each stakeholder because it will allow for a concerted effort towards implementation. Also crucial, and another strength that was highlighted, is strong leadership at all managerial levels (local, regional, and provincial) to drive the projects forward in the face of the numerous challenges. Another identified strength was the presence of a shared vision and philosophy from the community about what we want these programs to achieve, to guide the development of specific goals (everyone thinking and working together towards the same common goal).

#### Motivated clinicians working together

Other factors that facilitate the implementation include the availability of motivated clinicians, and immediate embracing of the program by some clinicians, and their willingness to go above and beyond what the pilot project was meant to implement. The presence of complete clinical teams composed of various professionals allows clinicians to learn from one another, and allows for strong clinical leadership within the diverse professions.

#### Evidence-based programs

Another important strength is the quality and the nature of the program structures themselves. While no two programs are identical, they are born out of one common evidence-based model, such as care being centered on the patient with a consideration of psychosocial and other risk factors, an emphasis on self-management, and a strong interdisciplinary component. There is therefore common ground for projects to learn from one another. In addition, there is an increased likelihood that stakeholders who subscribe to these principles will be motivated to implement the program. Of particular importance is the ability for a program to be able to weave a place for itself and share resources in a healthcare system that is lagging behind in the adoption of these chronic disease management principles. The ability of the programs in some regions to be able to integrate into existing structure was identified as an unexpected strength.

### Weaknesses to address

#### Insufficient resources

All groups indicated that the insufficiency of resources is an important challenge facing implementation. This includes lack of (1) funds on the local, regional, and provincial levels; (2) inadequate space to implement a given program the way in which it was originally intended; (3) a high turnover rate of the appropriate human resources occupying central roles within the program, and (4) short timelines. The timeline of the funding for the projects was identified as being too short to adequately implement the programs, and insufficient to be able to see significant clinical and/or behavioural changes in patients. Beyond having adequate resources for implementation, there needs to be enough resources to ensure a thorough evaluation and to carry this evaluation all the way through to the dissemination phase. In fact, the return of research results could ultimately be used to motivate all stakeholder groups, facilitate implementation, and bring about the desired changes of both current and future projects.

#### Organizational change within the clinical sites

Managing organizational changes, including resource re-allocation that is needed for full implementation of programs is one challenge that was raised more than once during the discussions and should not be underestimated. Within a single organization, there is the challenge of harmonizing different visions of chronic disease management. Organizational culture of an establishment can hinder implementation in situations where they do not coincide with a program’s philosophy, and become an even larger issue as programs begin to expand into larger territories. For example, health professionals who do not buy-into the concept of interdisciplinary care are less motivated to participate, which is of particular importance when that professional is needed as an integral part of the team. Even when everyone is open to change, it is a challenge to ensure that all professionals are applying their expertise to their full potential in the context of role transformation.

Another challenge identified is the need to balance the program and the elements of the CCM within the existing organisational structure, whose nature does not match with more concrete organization factors. An example being that the lack of remuneration of participating physicians can be perceived as a financial disincentive in the context of many of these chronic disease programs. Another related challenge is the potential resistance (by clinical teams) that can manifest itself when clinical practice and quality of care are being evaluated.

All organizational changes require strong leadership. Programs have a tendency to use “champion clinicians”; however, there is a danger in counting only on “champions” who are not always available. Further, a leader and a champion clinician play a distinct role. The leader’s function is to influence opinions of the members of the team, group, or establishment. The champion is asked to provide expertise and experience to peers regarding the innovative practices to be implemented. Implementation may be more successful if those planning the program think through who will play each of these roles, and if it will be the same person who will assume both roles. Also, if programs are relying on leaders who are too conservative, or lack the appropriate authority or influence to make the changes, this represents an additional weakness. Finally, creating organizational change is even more difficult with the high turnover of staff experienced by many of the clinical teams.

#### Lack of referrals from primary care physicians

The low number of referrals to the programs is an important challenge faced by many of the programs. Slow enrolment significantly affects the speed with which programs can be fully implemented. This is in part due to logistical problems, e.g. the fact that program referral forms are not ubiquitously available in referring physicians’ offices across a project’s territory. In some instances there may be a lack of clarity or knowledge of the referral process. It could also be that doctors, who have a more traditional approach to care delivery, might not yet be convinced of the efficacy of an interdisciplinary approach with such a large focus being placed on patient self-management. Many therefore signal the need for a communication or a marketing plan to be put in place to regularly promote the program to the target population and their treating health professionals. A communication strategy should outline the objective/goals of the communication, identify stakeholders, define key messages, pinpoint potential communication methods and vehicles for communicating information for a specific purpose, and specify the mechanisms that will be used to obtain feedback on the strategy [21]. The lack of human resources with the skills needed to support the selected communication strategy may only be one cause for low referring doctor engagement. Another cause might be that the communication procedure from the program to the referring doctor is difficult to fully implement (e.g. sending written feedback reports from the interdisciplinary team to the primary care physician to provide information about patient progress and outcomes). These communication strategies can be expanded to allow for a two-way communication that would allow doctors to contact the program to verify patient eligibility for the program or inquire about patient outcomes.

#### Program access

Aside from the challenges facing program implementation within an organization, it is also important to ensure that it is integrated into the existing healthcare system. The majority of projects require patients to have family doctors, but what will happen to the large proportion of the Quebec population who does not have one? In addition, and contrary to this problem of lack of access, participants highlighted that implementing a program might bring about a doubling of services that already exist on a territory; this needs to be avoided, as it would represent an inefficient use of resources.

### Theme 2: effectiveness of chronic disease management programs

Specific question for Theme 2: What possible opportunities are there for cross comparison of implementation and impact evaluation across the projects? Given the results of the projects to date, what are the remaining questions that need to be answered to determine the effectiveness and cost of implementing similar programs?

### Strengths to leverage

#### Engagement of stakeholders

A strong implication, a high level of motivation, and sincere interest on the parts of referring doctors, patients, clinicians, researchers and all other stakeholders facilitates the program evaluation as they all have a role to play in data collection, result interpretation, and dissemination. This engagement is maintained through a strong communication amongst all parties. Another strength that helps engage the less motivated referring physicians is when satisfied patients who see an improvement in their health convey this satisfaction to their family doctors thereby offering tangible evidence of the program’s effectiveness.

#### Learning from the similarities and differences between programs

The other major strength is the opportunity to inform the evaluation of the impact of the chronic disease management programs by considering the similarities and the differences between projects. The similarities allow for the sharing of common tools among multiple programs, which offers the possibility of comparing results and increasing sample sizes when evaluating impact on patient outcomes and primary care practice. While possible comparisons of results between projects should be explored further, caution is needed given the differences in patient populations and program objectives across projects. The different implementation strategies can be compared to identify the most effective components of these strategies.

### Weaknesses to address

#### Lack of referrals

Lack of referrals to the program, while hindering program implementation, also directly effects the evaluation by not having a large enough sample. This problem is compounded when participants leave the program early causing a situation of missing data. The slower the enrolment, the greater the delay the evaluation team has in being able to provide meaningful results. Many participants expressed the pattern whereby lack of participating patients tends to add to a difficulty in legitimizing and supporting the program’s advantages, and, in turn, family doctors are not encouraged to refer due to lack of evaluation results. In addition, it is often difficult to provide evaluation results to clinicians in a timely manner to support them in identifying ways to enhance the implementation of the programs and to assess the impact on patient care and to inform clinical decisions.

#### Barriers to program implementation

Many challenges affecting the implementation of programs were also evoked when participants discussed the evaluation theme. In addition to a lack of referrals to the program, others include: the difficulties in having all the appropriate resources (financial, human, space) necessary for implementation; a lack of access for patients who do not have a family doctor; cultural differences; and a short timeline for program implementation. In addition to this latter point is the consensus that evaluation timeframes are also too short to complete evaluations capable of showing meaningful results for dissemination.

#### Data collection

Data collection represents a significant challenge to demonstrating the program’s effectiveness and there are many factors contributing to this problem. The first is that drop outs by participating patients or clinicians before the completion of the program leads situations of missing data. Second, while the presence of such a comprehensive evaluation component can help with implementation of the programs by motivating stakeholders, if clinicians change their behaviour because they know they are being evaluated, it may also represent an important bias that will need to be accounted for in the interpretation of results. Nevertheless, these research projects are a unique opportunity for developing a culture of evaluation and improvement in primary care. The evaluation should therefore not be envisioned nor used as a “police” looking for mistakes and failures. Third is the fact that clinicians are being asked to play a central role in the data collections process which is something that is not traditionally part of their professional roles, therefore, there is sometimes a resistance or unwillingness to do so. Fourth is the lack of information technologies, which have the potential to greatly facilitate the data collection and management processes. In fact, if clinical information systems were present for all programs, it is likely that clinicians would see the burden of participating in data collection reduced.

#### Identifying indicators and appropriate control groups

A context in which many stakeholders have a voice in the selection of which data is to be collected was identified as a potential weakness. In fact, all stakeholders have different objectives, and want to measure different things. Therefore, the challenge is consolidating these needs and making decisions on which tools to use and which indicators to collect while keeping in mind the resource constraints of the evaluation component. Given the challenges to implementation and the large number of indicators being collected, the absence of a reference grid of minimum indicators to be collected by all programs is considered a weakness. By having established select required indicators, there would be an assurance of the minimum amount of information necessary to compare across projects.

Finally, given the nature of many of the projects, there are important challenges of finding the appropriate control groups needed to demonstrate a program’s effect.

### Common areas across Theme 1 and 2

The areas that were repeated in both Theme 1 and 2 included: Lack of funding for research and the need for additional resources to sustain implementation and evaluation over longer periods of time; Lack of patient referrals by primary care physicians; the short timeline for the program implementation and evaluation; the need for clinical information systems to facilitate data collection; communication between all stakeholders is needed to legitimize the project for stakeholders to facilitate implementation, and boost referrals; implementation and evaluation of the programs are easier when there are stakeholders that are engaged and interested with a common vision and goals. Finally the fact that all programs are based on the CCM, which is good for comparing implementation and possible outcomes across projects. The existence of these common themes highlights how closely a program’s implementation and its evaluation can be interdependent.

### Opportunities Theme 1 and 2

Given the influence that success factors and challenges for the implementation of the programs have on the evaluation component and vice versa, the opportunities expressed for both themes during the SWOT analysis are presented together.

#### Involvement of stakeholders and partners

There needs to be an effort to reinforce current partnerships and to cultivate new ones. Take referring doctors for example. More effort needs to be made to promote the programs and their advantages. Primary care physicians must be able to recognise the crucial role they play in the programs. If they feel more included they will perhaps be more willing to refer their patient’s to the programs.

More can also be done to grow community networks. Participants express a need to reach out to organizations that share a common vision and philosophy towards chronic disease management. This includes courting the participation of non-traditional partners such as YMCAs, pharmacists, fitness centers, etc.

Finally, the opportunity to develop a solid communication strategy that can be used to market the programs’ advantages to potential partners must be seized. Certain modalities are worth exploring, notably making use of marketing professionals or forming partnerships with universities to have marketing students develop a communication strategy for the programs. These partnerships could lead to the discovery of more effective ways to communicate evaluation results, which can help legitimize the programs and command the attention of potential partners and decision makers.

#### Seizing funding opportunities

Equally important for program implementation and evaluation is the need to remain vigilant for both traditional and alternative sources of public and private funding. This also involves capitalizing on existing funding to see how it may creatively be used to benefit chronic disease management programs. For example, there was recent provincial funding to add resources to FMGs. If aware of an influx of resources within an organization, programs can be designed and implemented in such a way as to capitalize on or share these resources thereby increasing overall efficiency within an organization.

#### Knowledge transfer

Perhaps the most widely expressed opportunity was the desire to facilitate the sharing of knowledge. The organization of knowledge translation events such as the one being discussed in this paper has been beneficial not only to provide a platform for the sharing of information and successful strategies but also in facilitating human contact between stakeholders. In addition, such events have the effect of propagating the synergy between the research and clinical teams, which was identified as one of the most important strengths to leverage. These events can occur more frequently and on a smaller scale by making use of existing resources such as local and regional tables to share and move ideas forward. By ensuring effective knowledge transfer, the current programs and future programs will be able to learn from each other and use clinical tools, experiences, and models of care which have already been proven effective. Finally, another opportunity for knowledge translation is ongoing synthesis and publication of facilitators and barriers of chronic disease management program implementation and evaluation, which can be used to inform current and future program planning.

#### Supporting the transformation of professional roles

As was discussed, one of the most important challenges is the transformation of professional roles. This is not surprising as it requires both changes within individuals and within organizational structures. Members from the clinical and evaluation teams alike offered a number of opportunities that could help support this change in professional roles: (1) review the remuneration of doctors as the current method does not coincide well the interdisciplinary format of care delivery; (2) explore the use of facilitators as opposed to relying on “medical champions” alone whom are not always available, facilitators may be in a better position to offer the intensity required to ensure transformation of professional roles; (3) bring professionals together to ensure the offering of identical training to ensure the standardization of care and more importantly the consistency of information being given to patients; (4) improve the working conditions of clinicians which will help incite clinician participation in the program and avoid the disruption caused when unsatisfied clinicians leave; (5) restructure the programs in such a way as to minimize the disruption caused by clinician turnover; (6) explore different types of professional training including courses on how to function within an interdisciplinary team and how to deliver interventions focused on patient self-management; (7) advocate for the reshaping of university curriculums to put greater emphasis on the advantages of interdisciplinary care and self-management, which may give future health professionals greater exposure to these concepts.

#### Health information technology

Expanding the use of health information technologies (HIT) in the healthcare system and in these programs was seen as an important opportunity. Many of the participants believe that HIT has the potential to facilitate or even solve many of the challenges currently facing these programs. For example: HIT can (1) facilitate the referral process into the program and help referring doctors follow their patient’s progress; (2) facilitate data collection to inform clinical decision making and the evaluation; (3) facilitate knowledge exchange which is important for the functioning of interdisciplinary teams, dissemination of evaluation results, and maintenance of positive stakeholder relations; and (4) improve the quality of a program’s interventions by providing access to more complete medical information and access to decisions support.

#### Cost evaluation

Given the allocation of funds specifically for the evaluation of these programs, many feel that this offers a rare opportunity to evaluate the costs involved in implementing the programs and the overall cost-effectiveness of these chronic disease management programs. This is a good opportunity that can supply invaluable information to decision making at the local, regional, and national level. One of the most important strengths for the evaluation of these programs is the similarity they have with one another, which allows for comparisons at many levels. However, a barrier to this comparison is that there were no minimum indicators identified for all programs to adhere to. Therefore, an opportunity would be the creation of a checklist of common indicators that would be used by all programs.

### Preliminary recommendations for knowledge transfer

The fact that the seven programs were all operating within a similar Quebec context enhanced the opportunity to discuss facilitators and barriers to implementation and evaluation. The following recommendations, based on the conclusions resulting from the presentations and discussions at the knowledge transfer meeting, are targeted to the local, regional, and provincial organizations involved in these programs. However, we believe that the results presented and the following recommendations are useful for organizations implementing similar programs, to provide a preliminary roadmap for organizing and delivering high-quality care for the prevention and management of chronic diseases.

#### Recommendations

- Dedicate resources and time to the promotion and advocacy of chronic disease prevention and management programs.
  - Promote the programs’ existence and advantages (by sharing clinical and evaluation data) with community health professionals in order to increase program referrals.
  - Reinforce current partnerships and cultivate new ones with community partners (e.g. fitness centers and pharmacists).
- Support the transformation of professional roles to be more in line with the current visions of chronic disease prevention and management programs.
  - To compare and evaluate the potential impact of different models of remuneration on the implementation of evidence-based practices
  - Conduct an environmental scan of the training programs in order to identify existing training programs, which put an emphasis on the role of professionals in the prevention and management of chronic disease.
  - Organize and offer continuing education training in interdisciplinary teamwork and patient self-management for primary care health professionals and interdisciplinary team members.
  - Provide training opportunities to support clinicians and clinical managers to adopt evaluation and feedback as part of their clinical process to reflect on current practices and enhance programs.
  - Advocate for more inclusion of chronic disease prevention and management principles in the curriculum of medical, nursing, and allied health programs in universities.
- Implement strategies to facilitate the implementation of chronic disease management programs.
  - Conduct an ongoing environmental scan of existing chronic disease management interventions and programs in each region to avoid duplication of services and maximize resources.
  - Provide human resources to help identify opportunities to maximize existing resources and facilitate organizational change during the implementation of new programs.
  - In addition to patient referral to chronic disease management programs by primary care physicians, provide referral mechanisms from other health professionals.
  - Ensure systematic support for self-management strategies.(e.g. motivational approach [22], the 5A approach [23]).
- Invest in the acquisition and the implementation of health information technologies to facilitate the standardized data collection of chronic disease prevention and management programs province-wide. These technologies should support and facilitate interdisciplinarity.
- Provide the resources needed to maximize the capacity and efficiency of implementing and evaluating chronic disease prevention and management programs.
  - Provide the financial resources required to sustain evaluations long enough to produce robust results on impact and cost-effectiveness.
  - Identify a minimum set of impact, process and implementation indicators that should be collected by all programs to facilitate comparisons of programs and impact on patient and primary care outcomes across projects.
- Dedicate financial and human resources for the planning of knowledge translation activities.

## Conclusion

Implementation of chronic disease management programs is complex. Comparison across programs currently being implemented provides the opportunity to identify common challenges and success factors for the delivery of interdisciplinary care for chronic conditions integrated into primary care. We do, however, caution that this represents a limitation for generalizing the results to other settings outside of Quebec. While many of the themes are in line with the literature on implementation theory, we recommend consulting a comprehensive theoretical framework, such as the Consolidated Framework for Implementation Research [24], to help guide the implementation of the presented recommendations in order to ensure relevance based on the setting.

Our synthesis across seven chronic disease programs identified the need to actively engage primary care physicians so that they may recognise the benefits and increase patient referral to the programs. In addition, mechanisms are needed to optimise access to chronic disease management programs and to avoid duplication of services. Further, working as an interdisciplinary team to support patients to help them develop the skills they need to take an active role in their care is new for many clinicians. A shift is needed in the way health professionals are trained to be more in line with the principles of chronic disease management. Within programs, technology will be key for facilitating the collection of clinical and patient reported data to feedback this information to inform clinical decision-making and to allow for ongoing evaluation and improvement of programs. Finally, a culture shift to drive home the importance of measurement and reflection of clinician practices is essential for ongoing improvement and sustainability of chronic disease management programs. A network for continuing knowledge translation between researchers, clinicians, patients, and decision makers will be necessary to speed up the implementation of best practice for chronic diseases by implementing strategies found to facilitate implementation.

## Additional files

10.1186/s13104-015-1514-0 Agenda of the *Fonds Pfizer-FRQS-MSSS sur les maladies chroniques* knowledge transfer meeting.
10.1186/s13104-015-1514-0 List of participants present at the *Fonds Pfizer-FRQS-MSSS sur les maladies chroniques* knowledge transfer meeting.

## Abbreviations

ASSS: health and social services agencies
CSSS: health and social services centres
MSSS: ministry of health and social services
CCM: Chronic Care Model
FRQS: Fond de recherche du Québec—Santé
FMG: family medicine group
SWOT: strengths, weaknesses, opportunities, threats
KT: knowledge translation
FMOQ: Fédération des médecins omnipraticiens du Québec
