# Supplementary material for: The prevention and management of chronic disease in primary care: recommendations from a knowledge translation meeting
Source: BMC Res Notes. 2015 Oct 15;8:571. doi: 10.1186/s13104-015-1514-0 (PMC4608115; doi:10.1186/s13104-015-1514-0)
Supplement: Supplementary file 1 — 10.1186/s13104-015-1514-0 Agenda of the Fonds Pfizer-FRQS-MSSS sur les maladies chroniques knowledge transfer meeting. [file 13104_2015_1514_MOESM1_ESM.pdf]

Additional File 1: Agenda

ORDRE DU JOUR	PLAGE HORAIRE
**Déjeuner/Accueil**	8 :00 - 8 :30
**Mot de bienvenue et présentation de la journée**<br>1. FRQS : Dr Renaldo Battista<br>2. Pfizer : Patrick Roy<br>3. MSSS : Dr Antoine Groulx : Présentation des animateurs de la journée	8 :30 - 8 :45
**Présentation de la journée**<br>Animateurs : Dr Galarneau et Dr Ricard<br>• **Objectifs de la journée** :<br>◦ Cette journée a comme objectif de partager les résultats obtenus par les sept équipes de décideurs-chercheurs ayant été subventionnées dans le cadre de ce programme et de discuter des moyens à déployer pour transférer ces connaissances aux différents CSSS, et ainsi maximiser l'utilisation des données probantes issues du contexte québécois. Cela permettra de soutenir la planification et l'organisation des services de première ligne destinés aux personnes atteintes de maladies chroniques.<br>• **Déroulement de la journée** :<br>◦ En avant-midi : présentation des 7 projets subventionnés<br>◦ En après-midi : discussion en tables rondes afin d'identifier les forces et faiblesses de chacun des projets et voir certaines similitudes entre les différents projets	8 :45 - 8 :55
**Présentation des projets**<br>Animateurs : Dr Galarneau et Dr Ricard<br>• Présentation de chacune des équipes :<br>• animation de la séance de questions (5 minutes avant la fin de chacune des présentations)<br>◦ avant la pause : Dr Ricard<br>◦ après la pause : Dre Galarneau	
**Mise en œuvre et évaluation d'un réseau intégré de prévention et de la gestion de la douleur chronique en 1ère ligne**<br>Chercheurs subventionnés: Sara Ahmed/Mark Ware<br>Présentateurs :	8 :55 - 9 :25
**Mise en oeuvre d'un réseau intégré de prévention et de gestion du risque cardiométabolique en 1ère ligne à Montréal**<br>Chercheurs subventionnés: Dr Pierre Tousignant/Dre Johanne Desforges<br>Présentateurs : Christiane Barbeau, Dominique Grimard et Dre Sylvie Poirier	9 :25 - 9 :55

**TRANSIT** - **Programme de TRANSformation des pratiques cliniques InTerprofessionnelles pour améliorer la qualité des soins préventifs cardiovasculaires en 1ère ligne**<br>Chercheurs subventionnés: Dr Alain Turcotte/Lyne Lalonde<br>Présentateurs :	9 :55 - 10 :25
**SIID2** - **Suivi intersectoriel et interdisciplinaire du diabète de type 2**<br>Chercheurs subventionnés: Dr Sylvère Vandemoortele/Dre Maryse Guay<br>Présentateurs : Dre Maryse Guay, Lise Jetté, Linda Lanthier	10 :25 - 10 :55
**Pause**<br>Animateurs : Dr Galarneau et Dr Ricard<br>• Amener les gens vers la pause	10 :55 - 11 :10
**Pr1MAC - Implantation et évaluation d'une intervention d'intégration des services de réadaptation en maladies chroniques aux soins de 1ère ligne**<br>Chercheurs subventionnés: Dr Martin Fortin/ Dr Martin Bélanger<br>Présentateur : Dr Martin Fortin	11 :10 - 11 :40
**V1sages** - **Évaluation et implantation pragmatique d'une intervention de gestion de cas et de soutien à l'autogestion pour les personnes atteintes de maladies chroniques vulnérables en 1ère ligne**<br>Chercheurs subventionnés: Dr Catherine Hudon/Maud-Christine Chouinard<br>Présentateurs :	11 :40 - 12 :10
**La prise en charge de sa santé sur le territoire de Rocher-Percé**<br>Chercheurs subventionnés: Stella Travers/Josée Gauthier<br>Présentateurs :	12 :10 - 12 :40
**Synthèse de l'avant-midi**<br>Animateurs : Dr Galarneau et Dr Ricard<br>• Réaction spontanée<br>• Lien entre les présentations de l'avant-midi et la discussion de l'après-midi<br>• Inviter les gens à aller au buffet dans la salle	12 :40 - 12 :45
**Lunch dans la salle**	12 :45 - 13 :40
**Explications du déroulement de l'après-midi**<br>Animateur : Dr Ricard<br>• Expliquer la séance de l'après-midi et logistique (salles)<br>○ Discussion en tables rondes sur 2 thèmes<br>○ Désigner un facilitateur et un secrétaire par table<br>○ Thème 1 dans la salle plénière (James McGill)<br>○ Thème 2 dans la salle Charles de Bleury au 11ème étage (Manon va les y conduire)<br>• Mentionner les 2 thèmes de discussion et les attentes de la discussion en tables rondes qui sont: répondre aux questions avec la participation de	13 :40 - 13 :45

différentes équipes et représentants des agences et présenter une synthèse après la pause

Thème 1 : Facteurs de réussite et défis de la mise en œuvre des programmes de gestion des maladies chroniques

Thème 2 : Efficacité des programmes de gestion des maladies chroniques

- Après la pause, chacun des facilitateurs (7 au total) devra venir présenter une synthèse des discussions (10 minutes)

Discussions en tables rondes 13 :45 - 15 :05

Animateurs : Dr Galarneau et Dr Ricard

- Rôle de facilitateur pour chacun des thèmes assignés
- Dre Galarneau pour le thème 1
- Dr Ricard pour le thème 2

Pause : 15 :05 - 15 :20

Discussion du comité organisateur et des animateurs afin de déterminer la direction à prendre à-propos des projections pour la 2ème journée
(15 minutes d'échanges, dirigé par Sara Ahmed)

Partage des discussions de chacun des groupes : 10 min/table 15 :20 - 16 :30

Animateurs : Dr Galarneau (animation) et Dr Ricard (prise de notes)

Résumé de la journée et projections pour la 2ème rencontre 16 :30 - 16 :55

Animateurs : Dr Galarneau et Dr Ricard

- 10 minutes à 2 pour une vision globale des discussions
- 15 minutes d'échange avec la salle, principalement par rapport aux projections pour la 2ème rencontre :
  - Cette 2ème partie (15 minutes) sera dirigée par Sara Ahmed, chercheure subventionnée, et membre du comité organisateur. Selon le déroulement de la journée, nous discuterons à la pause de la direction que devrait prendre cette dernière partie de la journée.

Fin de la rencontre 16 :55 - 17 :00

FRQS : Anne-Cécile Desfaits
