# Supplementary material for: The prevention and management of chronic disease in primary care: recommendations from a knowledge translation meeting
Source: BMC Res Notes. 2015 Oct 15;8:571. doi: 10.1186/s13104-015-1514-0 (PMC4608115; doi:10.1186/s13104-015-1514-0)
Supplement: Supplementary file 2 — 10.1186/s13104-015-1514-0 List of participants present at the Fonds Pfizer-FRQS-MSSS sur les maladies chroniques knowledge transfer meeting. [file 13104_2015_1514_MOESM2_ESM.pdf]

Additional File 2: List of participants

Nom	Poste/Projet	Organisation
**Région 01 - Bas St-Laurent**		
Dominique Perron	Professionnel responsable - Dossier Maladies Chroniques	ASSS Bas St-Laurent
Marc St-Laurent	Directeur régional des ressources informationnelles, Évaluateur du Programme Pfizer-FRQS-MSSS Maladies Chroniques	ASSS Bas St-Laurent
**Région 02 – Saguenay-Lac St-Jean**		
Dr Martin Bélanger	PR1MAC, chercheur principal	ASSS Saguenay-Lac-St-Jean
Dr Tarek Bouhali	PR1MAC, coordonnateur	CSSS Chicoutimi/Université de Sherbrooke
Bayéro Diallo	PR1MAC	CSSS Chicoutimi
Dr Martin Fortin	PR1MAC, chercheur principal	CSSS Chicoutimi/Université de Sherbrooke
Maud-Christine Chouinard	V1sages, chercheure principale	CSSS Chicoutimi/UQAC
Dre Catherine Hudon	V1sages, chercheure principale	CSSS Chicoutimi/Université de Sherbrooke
Martine Couture	Présidente Directrice Générale	ASSS Saguenay-Lac-St-Jean
Dr Donald Aubin	Dir. Services de santé, affaires médicales, universitaires et hospitalières	ASSS Saguenay-Lac-St-Jean
Katia Tousignant	Professionnelle responsable - Dossier Maladies Chroniques	ASSS Saguenay-Lac-St-Jean
**Région 03 – Capitale Nationale QC**		
Hélène Bélanger	Professionnelle responsable - Dossier Maladies Chroniques	ASSS Capitale Nationale (QC)
**Région 04 – Mauricie et Centre du QC**		
Gilles Hudon	Directeur des services de santé et des affaires médicales	ASSS Mauricie et Centre du QC

Nom	Poste/Projet	Organisation
Josée Massicotte	Professionnelle responsable - Dossier Maladies Chroniques	ASSS Mauricie et Centre du QC
**Région 05 – Estrie**		
Lynda Périgny	Directrice des services, des affaires médicales et universitaires	ASSS Estrie
Audrey-Anne Simard	Professionnelle responsable - Dossier Maladies Chroniques	ASSS Estrie
**Région 06 – Montréal**		
Sara Ahmed	Chercheure principale (subventionnée)	MUHC - Hôpital Royal Victoria/Université McGill
Tim Halal	Physiothérapeute	CSSS Cavendish
Marie-Andrée Lahaie	Psychologue	CSSS Ouest de l'Île/Cavendish
Susan Templeman	Infirmière	CSSS Vaudreuil-Soulanges
Regina Visca	Coordonnatrice	RUIS McGill
Dr Mark Ware	Chercheur principal (subventionné)	Hôpital Général de Montréal/Université McGill
Dr André Bélanger	Médecin conseil	ASSS Montréal
Dre Johanne Desforges	Chercheure principale (subventionnée)	CSSS Sud-Ouest-Verdun
Dominique Grimard	Agente de planification, de programmation et de recherche Services préventifs en milieu clinique	Santé publique Montréal
Dr Pierre Larochelle	Médecin conseil	ASSS Montréal
Dre Sylvie Provost	Médecin conseil	Santé publique Montréal
Dr Pierre Tousignant	Chercheur principal (subventionné)	Université McGill
Christiane Barbeau	Coordonnatrice en Gestion de maladies chroniques	ASSS Montréal
Dr Richard Bergeron	Adjoint médical au directeur des services généraux et des maladies chroniques	ASSS Montréal
Henriette Bilodeau	Professeure - École des Sciences de la Gestion	UQAM

Nom	Poste/Projet	Organisation
Gilles Dupuis	Directeur scientifique du Centre de liaison sur l'intervention de la prévention psychosociale	CLIPP/UQAM
Dr Rod Finlayson	Directeur	RUIS McGill Centre d'Expertise pour la douleur chronique
Shandi Miller	Coordonnatrice Réseau-1 Québec, Projets spéciaux, Centre de recherche	Centre hospitalier St-Mary
Dr Jacques Ricard	Directeur des services généraux et des maladies chroniques, Animateur	ASSS Montréal
**Région 07 – Outaouais**		
Marilyne Côté	Chef de l'Administration des Programmes, Services Généraux de Santé	ASSS Outaouais
Julie Larivière	Professionnelle responsable - Dossier Maladies Chroniques	ASSS Outaouais
**Région 08 – Abitibi-Témiscamingue**		
Dr Éric Lampron-Goulet	Adjoint médical au directeur de santé publique	Direction Santé Publique Abitibi-Témiscamingue
**Région 10 – Baie-James**		
Vincent Rajotte	Professionnel responsable - Dossier Maladies Chroniques	ASSS Baie-James
**Région 11 – Gaspésie-Îles de la Madeleine**		
Josée Gauthier	Chercheure principale (subventionnée)	INSPQ/UQAR
Tim Sutton	Kinésiologue	CSSS du Rocher-Percé
Stella Travers	Chercheure principale (subventionnée)	CSSS du Rocher-Percé
Dre Yolaine Galarneau	Présidente Directrice Générale, Animatrice	ASSS Gaspésie-Îles de la Madeleine
**Région 12 – Chaudière-Appalaches**		

Nom	Poste/Projet	Organisation
France Tanguay	Professionnelle responsable - Dossier Maladies Chroniques	ASSS Chaudière-Appalaches
**Région 13 – Laval**		
Céline Bareil	TRANSIT co-chercheure	HEC Montréal
Dre Eveline Hudon	TRANSIT co-chercheure	Université de Montréal
Lyne Lalonde	TRANSIT chercheure principale	CSSS Laval/Université de Montréal
Lise Lévesque	TRANSIT coordonnatrice	CSSS Laval
Dre Marie-Thérèse Lussier	TRANSIT co-chercheure	CSSS Laval/Université de Montréal
Dr Alain Turcotte	TRANSIT chercheur principal	CSSS Laval
Maria Mata	Professionnelle responsable - Dossier Maladies Chroniques	ASSS Laval
**Région 14 - Lanaudière**		
Maryse Tremblay	Professionnelle responsable - Dossier Maladies Chroniques	ASSS Lanaudière
**Région 15 - Laurentides**		
Rolande Daignault	Consultante	ASSS Laurentides
**Région 16 - Montérégie**		
Dre Monia Ghorbel	Résidente 3 santé publique et médecine préventive, stagiaire à la direction de santé publique de la Montérégie (SIID2)	Université de Sherbrooke/ CSSS Champlain Charles-Le-Moyne
Dre Maryse Guay	SIID2 chercheure principale	Université de Sherbrooke/ASSS Montérégie
Lise Jetté	SIID2 Infirmière clinicienne	CSSS Champlain Charles-Le-Moyne
Linda Lanthier	SIID2 Nutritionnniste	CSSS Champlain Charles-Le-Moyne
Sarah Dutilly-Simard	Agente de planification, de programmation et de recherche Secteur planification, évaluation et recherche	Direction de Santé Publique, Montérégie

Nom	Poste/Projet	Organisation
Dre Sylvie Tardif	Directrice des affaires médicales, universitaires et des partenariats	ASSS Montérégie
Liliane Thystere	Professionnelle responsable - Dossier Maladies Chroniques	ASSS Montérégie
**Pfizer Canada**		
Farzad Ali	Directeur « outcomes research »	Pfizer Canada
Ashley Coughlin	Directeur de marque	Pfizer Canada
Denise Cloutier	Directrice, stratégies et politiques de la santé au sein de la division des affaires publiques et des relations	Pfizer Canada
Sophie Rochon	Chef, Remboursement et politiques de la santé – QC	Pfizer Canada
Patrice Roy	Directeur, Recherche et développement, Québec et Atlantique	Pfizer Canada
**FRQS**		
Dr Renaldo Battista	Directeur Scientifique	FRQS
Anne-Cécile Desfaits	Directrice des programmes (intérim) et des partenariats	FRQS
Karine Genest	Chargée de programmes	FRQS
Manon Pelletier	Chargée de programmes- Maladies Chroniques	FRQS
**MSSS**		
François Dubé	Directeur adjoint de l'organisation des services de 1ère ligne intégrés	MSSS
Francis Dubois	Conseiller en orientation et coordination de la recherche et de l'innovation – Direction de la recherche, de l'innovation et du transfert de connaissances	MSSS
Rana Farah	Professionnelle responsable - Dossier Maladies Chroniques	MSSS
Dr Antoine Groulx	Directeur de l'organisation des services de 1ère ligne intégrés	MSSS
Geneviève Landry	Coordonnatrice en développement et gestion de projets - Direction de l'organisation des services de 1ère ligne intégrés	MSSS

Nom	Poste/Projet	Organisation
Jacques Rhéaume	Conseiller en orientation et coordination de la recherche et de l'innovation – Direction de la recherche, de l'innovation et du transfert de connaissances	MSSS
